# The Role of Threats in the Support of System-Justifying Beliefs

**DOI:** 10.11621/pir.2025.0207

**Published:** 2025-06-01

**Authors:** Irina S. Prusova, Anna S. Gorokhova

**Affiliations:** a HSE University, Moscow, Russia

**Keywords:** economic system justification, fear of death, economic threats, belief in a dangerous world, belief in a competitive world

## Abstract

**Background:**

Encounters with threats can lead to a motivation to justify the existing social system, which can be expressed through endorsement of system-justifying beliefs.

**Objective:**

The aim of the study was to examine how difierent types of threats contribute to endorsement of system-justifying beliefs in the economic domain.

**Design:**

We tested a theoretical model (*N* = 577) with internal threats (fear of death), economic threats (threats of poverty and socio-economic inequality), and subjective threat perception (belief in a dangerous and competitive world) as predictors; system-justifying beliefs (economic system justification, opposition to equality, dominance, and antiegalitarianism) served as dependent variables, and sociodemographic characteristics were included as control variables.

**Results:**

Structural equation modeling showed adequate fit in the Russian context. Belief in a competitive world positively predicted dominance, antiegalitarianism, and opposition to equality, while belief in a dangerous world negatively predicted economic system justification. Fear of death positively predicted opposition to equality, whereas perceived economic threats (poverty and inequality) negatively predicted antiegalitarianism, and opposition to equality.

**Conclusion:**

Dangerous and competitive worldviews, internal and economic threats contribute to the support of system-justifying beliefs in the economic domain.

## Introduction

Crises and global transformations repeatedly arise within a multitude of nation states and afiect their citizens’ socioeconomic well-being. In recent years, Russians have had to face the epidemiological impact of COVID-19 and consequences of geopolitical changes. At the macroeconomic level, the economic crisis has led to rising levels of inequality and inflation, and a decrease in GDP (Rosstat, 2023). One might assume these changes would elicit threats to social and economic well-being, for example, the deterioration in living standards, loss of savings, and uncertain decision-making. The context of crises and geopolitical changes influences the perception of stability and predictability of the world (Jetten et al., 2017). Public polls showed that in 2023, Russians mostly experienced anxiety about international conflicts (23%), socio-economic inequality (22%), and low incomes (13%) (VCIOM, 2024a). At the same time, polls showed that the share of Russians who overestimated their economic status (23%) was higher in comparison to those who underestimate it (17%) (FOM, 2024). This perspective was observed at both the individual and national levels. Previous studies indicated that during the period of the COVID-19 pandemic people tended to endorse the economic system, such as the Russian economy (for example, the general economic situation and production in particular) (Agadullina et al., 2021). Around 57% of Russians claimed that Russia is *better than other countries*, which represents an increase compared to previous years (VCIOM, 2023). Furthermore, 56% of Russians noted that the economic sanctions imposed in recent years had no impact on their lifestyle (FOM, 2023).

The readiness to endorse the prevailing economic system while under perceived threat is reflected in System Justification Theory (SJT) (Jost & Banaji, 1994). Jost et al. (1994) observed that there exists a tendency in people to justify a society’s economic system in those societies where there exists extreme economic inequality. The maintenance of the status quo and the endorsement of hierarchy may manifest in system-justifying beliefs, such as political conservatism (resistance to change and acceptance of inequality; Jost et al., 2003), right-wing authoritarianism (RWA) (tendency to submit to established authorities, adherence to traditional social norms and aggression towards norm violators; Altemeyer, 1996), system justification (belief that inequality and the status quo are legitimate and inevitable; Jost & Banaji, 1994), opposition to equality (perception of equality as an undesirable principle of an economic system reflected in support for unequal income distribution; Kluegel & Smith, 1986), social dominance orientation (SDO) (support for a group-based hierarchy and intergroup inequality; Pratto et al., 1994).

### Threats and System-Justifying Beliefs

Jost et al. (2003) suggested that support for system-justifying beliefs might satisfy the existential needs to reduce perceived threats and regain the perception of control. In order to analyze the role of threats, Jost et al. (2017) distinguished between objectively threatening situations (societal sources of threat linked to situational changes, e.g., terror attacks, war, poverty, economic crisis, socio-economic inequality), subjective threat perceptions (beliefs in a dangerous and a competitive world), and internal threats (threat related to an individual’s private life, *e.g*., fear of death and death anxiety). The results of the meta-analysis showed that objectively threatening situations, subjective perception of threat, and internal threats show significant effects on the endorsement of the system-justifying beliefs (Jost et al., 2003).

### Internal threats

Internal threats (fear of death or mortality salience) enhanced the support for system-justifying beliefs (Jost et al., 2017). Fear of death is positively linked to economic attitudes in the US (Hennes et al., 2012) and the Netherlands (Onraet et al., 2013), conservatism on the ideological self-placement in the US (Jost et al., 2017), and dominance (a subscale of SDO) in Russia (Prusova et al., 2022). However, some studies found that the effect of fear of death vary across difierent system-justifying beliefs. Fear of death was strongly associated with dominance (i.e., support for a group-based hierarchy in which high-status groups oppress and control low-status groups), but not with antiegalitarianism (i.e., support for inequality between groups) (Prusova et al., 2022). Preference for hierarchy may be linked to perceived order in the world, in which high-status group members may protect both high status and low status group individuals from threatening conditions (Cohen et al., 2017).

In addition to fear of death, experimental studies researchers have primarily focused on the effect of mortality salience (Jost et al., 2017). Mortality salience reinforced the *conservative shift*, such as increased support for conservatives’ political figures and programs, as well as a tendency toward conservative self-placement on the ideological continuum (Jost et al., 2017). In contrast, meta-analysis showed that instead of a *conservative shift,* six studies indicated a *liberal shift* and 24 studies showed no significant effect (Jost et al., 2017). Based on terror management theory (TMT), support for contradictory orientations might be explained by the *cultural worldview polarization hypothesis* (Burke et al., 2013). More specifically, mortality salience might enhance pre-existing views causing liberals to be more liberal, while conservatives become more conservative. For instance, a detailed study observed a more pronounced liberal shift in response to threat among a sample group in which participants held existing liberal views (Castano et al., 2011).

### Subjective Perceptions of Threat

Subjective perceptions of threat, such as dangerous and competitive worldviews, have been positively associated with RWA, SDO, economic system justification, and political conservatism (Jost et al., 2017). This effect was confirmed in 112 out of 186 tests conducted on samples from the Netherlands, Belgium, the USA, South Africa, Canada, Sweden, France, and Italy (Jost et al., 2017). However, difierent types of threat might induce specific responses in support of system-justifying beliefs. For instance, in Russia difierent dimensions of opposition to equality were associated with difierent social worldviews. Belief in a dangerous world was positively linked to approval of equality and had an insignificant association with approval of inequality, whereas belief in a competitive world was negatively linked to approval of equality, but positively associated with approval of inequality (Sychev & Protasova, 2020). In line with the dual-process motivational model (DPM) of ideology, the perception of the world as a fearful and chaotic place (*i.e.*, belief in a dangerous world) predicts right-wing authoritarianism, whereas perception of the world as a place of struggle for resources (*i.e.*, belief in a competitive world) enhances social dominance orientation (Duckitt et al., 2002).

### Objectively threatening situations

Objectively threatening situations, such as terror attacks or war, were positively associated with conservative and right-wing shifts in Spain, Israel, the UK, Germany, and the USA (Jost et al., 2017). An analysis of country-level threats showed broad correlation with observable factors such as declining gross national product, inflation, increasing unemployment, decreases in life expectancy, and rising homicide rates in 91 nations. This showed a similar effect linking support for right-wing orientation in social-cultural (.70) and economic-hierarchical (.79) attitudes (Onraet et al., 2013). In Russia, the threat of terrorism enhanced a rightward shift in support for control in the economic domain (Prusova & Gulevish, 2020). However, in Germany, similar external threats, such as terror attacks, correlate with a pronounced left-wing shift among leftists (Thörner, 2014). These differing tendencies among rightist and leftists might be explained by difierent mechanisms underlying the previously discussed ideologies under external threat. For example, rightists might wish to have a strong leader, whereas leftists might worry about political leaders abusing their increased power (Thörner, 2014).

Studies have indicated that system-justifying beliefs can be interchangeable. However, the means and mechanisms they employ to address inequality and mitigate the effects of threat can differ (Jost et al., 2017). Therefore, difierent types of threat might induce specific and non-specific responses in the support of system-justifying beliefs, which are mostly represented in the sphere of political preferences and intergroup relations (Jost et al., 2017). In addition, studies that provide analysis of objectively threatening situations and their effects have mostly focused on dynamics of intergroup threats such as war or terror attacks, without providing detailed insight into the effects of economic threats such as poverty or inequality (Jost et al., 2017). According to polls, in 2023 Russians were most afraid of socio-economic threats such as inequality and low income (VCIOM, 2024a). Therefore, the range of responses to and perceptions of existing threats elicit questions concerning their influence in swaying opinions concerning the support of system-justifying beliefs in the economic domain.

### The current research

The aim of the current study was to examine the role of economic threats, fear of death, and dangerous and competitive worldviews in the endorsement of system-justifying beliefs in Russia. The current study extends the SJT framework, providing a more detailed analysis of the existential motivational foundations. In a previous meta-analysis, Jost et al. (2017) defined these existential motivational foundations as a combination of internal threats, objectively threatening situations, and subjective perceptions of threat. However, the previously outlined *types of threat* differ in their effect on system-justifying beliefs (*r_internal_* = .08–.13; *r_subjective_* = .12–.31; *r_objective_* = .07–.14). Moreover, in line with the model of political conservatism as motivated social cognition, previous studies primarily focused on the multifaceted nature of political conservatism, while often overlooking system-justifying beliefs in the economic sphere (Jost et al., 2003). In the present study, we analysed the independent effect of internal and objective threats, and subjective perceptions of threat within one comprehensive model, incorporating difierent system-justifying beliefs within the economic domain.

We tested a model in which economic threats (poverty and socio-economic inequality), fear of death, belief in a dangerous world and belief in a competitive world were considered predictors; system-justifying beliefs - economic system justification, opposition to equality, dominance and antiegalitarianism (SDO subscales) - were included as dependent variables, and socio-demographic characteristics were included as control variables. Conservatism was not included in the current model as a system-justifying belief because, in post-Soviet countries, the conservatism-liberalism dichotomy did not represent the specifics of political views in the social and economic spheres (Aspelund et al., 2013). The choice of control variables was based on evidence suggesting that the motivation to bolster the status quo and inequality might depend on income level, education, subjective social status, and gender (Vargas-Salfate et al., 2018).

### Social Context of the Study

Previous studies were primarily conducted in WEIRD (Western, Educated, Industrialized, Rich, and Democratic) contexts, with fewer studies in non-WEIRD settings such as those with a history of socialism. The results showed that system-justifying beliefs were stronger in the WEIRD countries compared with Eastern Europe (Cichocka & Jost, 2014). This discrepancy in system justification might be explained by differing societal norms with meritocracy being prevalent in traditionally capitalist countries, and egalitarianism in post-communist countries (Smith & Mateju, 2012). However, in post-Soviet contexts, these principles of justice showed much lower internal consistency than in WEIRD settings. For instance, in Russia people simultaneously support contradictory principles of the capitalist market (meritocratic beliefs) and socialist welfare (egalitarian beliefs) (Smith & Mateju, 2012).

During the collapse of the Soviet Union, the transition to capitalism and democracy was associated with high social and economic costs, including high rates of inflation, unemployment, socio-economic inequality, and poverty (Smith & Mateju, 2012). The economic crises of the 1990s reinforced the perception of economic inequality, corruption, and the unfairness of market-based principles within post-Soviet countries (Smith & Matějů, 2012). To deal with economic insecurity, people may demonstrate support for governmental regulation in difierent spheres of social life, such as reducing the income gap between citizens (VCIOM, 2022). Public polls showed that Russians demonstrated a high level of trust towards political institutions (VCIOM, 2024b). These indicators reflect the prevalence of system-justifying beliefs that serve a palliative function, mitigating negative afiect. However, according to public polls conducted in recent years, Russians were most afraid of socio-economic threats, including socio-economic inequality, a decline in income, and rising prices for common goods (VCIOM, 2024a). Therefore, these results prompt salient research questions concerning the role of perceived threats in system-justifying beliefs within the Russian economic sphere.

## Methods

### Participants

For the online study, 747 participants were recruited through Yandex.Toloka (an Amazon MTurk equivalent) and were paid $.08 each for their participation. We used the maximum longstring method to detect careless responding and excluded participants who demonstrated the same response to more than six consecutive items in the questionnaire (Niessen et al., 2016). We also excluded participants who failed to finish the survey. After the selection procedure, the final sample included 577 Russians (52% female), aged between 18 and 74 years old (*M_age_* = 37.1; *SD_age_* = 11.0). Approximately 46.3% (*n* = 267) of participants had university degrees, 90.6% (*n* = 523) identified themselves as Russians and around 50% (*n* = 272) identified as religious.

### Procedure

The online study was conducted in May 2023. We developed an online questionnaire using the SurveyMonkey platform. At the beginning of the study, participants completed the informed consent form, and thereafter, they completed questionnaires on the fear of death, dangerous worldview, competitive worldview, economic threats (poverty and socio-economic inequality), economic system justification, social dominance orientation, opposition to equality, and socio-demographic questions.

#### Questionnaires

*Belief in a Dangerous World*. To measure the perception of the world as an insecure and unpredictable place, we used the *Russian version of Duckitt et al.’s (2002) Belief in a Dangerous World scale* (Cronbach’s *α* = .84) (Gulevich et al., 2014). Participants assessed the degree of agreement with 5 items (*e.g*., “There are many dangerous people in our society who will attack someone out of pure meanness, for no reason at all”) on a 7-point Likert Scale from 1 (strongly disagree) to 7 (strongly agree).

*Belief in a Competitive World.* To measure the perception of the world as a ruthless struggle for resources and power, we used the *Russian version of Duckitt et al.’s (2002) Belief in a Competitive Worldview Scale* (Cronbach’s *α* = .79) (Gulevich et al., 2014). Participants assessed their agreement with five items (*e.g.*, “If it’s necessary to be cold-blooded and vengeful to reach one’s goals, then one should do it”) on a 7-point Likert scale from 1 (strongly disagree) to 7 (strongly agree).

*Fear of Death.* To measure the fear of death, we used the *Russian version of the Wong et al.’s (1994) subscale* (Cronbach’s *α* = .92) (Chistopol’skaya et al., 2017). Participants assessed the degree of agreement with four items (*e.g*., “The prospect of my own death arouses anxiety in me”) about feelings of concern related to death on a 7-point Likert scale from 1 (strongly disagree) to 7 (strongly agree).

*Economic Threats (Poverty and Socio-Economic Inequality).* To measure the economic threat of poverty (Cronbach’s *α* = .88) and socio-economic inequality (Cronbach’s *α* = .89), we used the *Financial Threat Scale*, which was adapted to Russian in the pilot study (Cronbach’s *α* = .89) (Marjanovic et al., 2013). Participants were asked to think about poverty and socio-economic inequality and thereafter report how often they thought about these threats and to which degree they felt threatened by and concerned about them on a 5-point Likert Scale from 1 (not at all) to 5 (very much).

*Economic System Justification.* To measure the motivation to justify the economic system, we used the *Russian version of Jost and Thompson’s (2000) Economic System Justification scale* (Agadullina et al., 2021). Participants evaluated their degree of agreement with six statements (Cronbach’s *α* = .80; *e.g*., “In Russia today, government control of resources is necessary to support the economy”) on a 9-point Likert scale from 1 (strongly disagree) to 9 (strongly agree).

*Social Dominance Orientation.* To measure SDO, we used the *Russian version of the Pratto et al.’s (1994) Social Dominance Orientation Scale* (Gulevich et al., 2018). SDO included two subscales: Dominance (five items; Cronbach’s *α* = .77; *e.g*., “It’s probably a good thing that certain groups are at the top and other groups are at the bottom”) and antiegalitarianism (five items, Cronbach’s *α* = .78; “Group equality should be our ideal”). Participants evaluated the degree of agreement with ten statements on a 7-point Likert scale from 1 (strongly disagree) to 7 (strongly agree).

*Opposition to equality.* To measure the support of unequal income distribution, we used the *Russian version of the Inequality subscale from Kluegel and Smith’s (1986) Beliefs About Inequality Scale*, which was adapted in a preliminary study. Participants evaluated the degree of agreement with four items (Cronbach’s *α* = .88; *e.g*., “Incomes should not be made more equal since that would keep people from dreaming of someday becoming a real success”) on a 7-point Likert scale from 1 (strongly disagree) to 7 (strongly agree).

*Subjective Socioeconomic Status.* To measure subjective socioeconomic status (SSES), we used a socioeconomic ladder (“Where would you place yourself on this ladder?” 1= the lowest level; 10 = the highest level) (Adler et al., 1994). Participants rated their position on the ladder on a scale from 1 (“closer to people who find themselves at the bottom”) to 10 (“closer to people who are at the top of the ladder”).

*Objective Socioeconomic Status.* To indicate objective socio-economic status (OSES), we asked participants to rate their income level on a scale from 1 (“There is not enough money even for food”) to 6 (“I can afford everything”).

*Education.* To measure education, we asked participants to choose their attained level of education from seven given options: elementary education, secondary education (school), specialized secondary education (college), incomplete higher education (currently enrolled in a bachelor’s/specialty programme), complete higher education, PhD or two or more degrees in higher education.

### Data processing

To examine the role of economic threats, fear of death, dangerous and competitive worldview in the support of system-justifying beliefs, we conducted a Spearman’s rank correlation analysis using Jamovi 2.3.28 (The Jamovi Project, 2024) and structural equation modeling (SEM) using the “lavaan” package (Rosseel, 2012) in RStudio software (RStudio Team, 2020). We used the following indices and criteria to estimate acceptable model fit: *χ*^2^ index, the Root-Mean-Square Error of Approximation (*RMSEA* < .08), Comparative Fit Index (*CFI* > .90) the Tucker-Lewis index (*TLI* > .90), and Standardized Root Mean Square Residual (SRMR < .08) (Schreiber et al., 2006).

### Links to Dataset with Codes

The datasets with codes, with detailed information about the variables, are available in the Open Science Framework repository: https://osf.io/6xm3q/?view_only=8548448ddc9c45278d4e78f0f78d4902

## Results

*Table 1* contains the descriptive statistics, Cronbach’s α, and the correlations between the variables. The results of the correlation analysis correspond to Jost’s, (2021) empirical findings. Economic system justification, dominance, and opposition to equality were primarily positively intercorrelated. In contrast, antiegalitarianism was negatively linked to economic system justification, but positively linked to the opposition to equality and dominance.

**Table 1:** Descriptive statistics, Cronbach’s α, and correlations between the variables

Variables	M	SD	α	1	2	3	4	5	6	7	8	9	10	11	12	13
1 Economic system justification	5.53	1.69	.80	—												
2 Dominance	3.06	1.24	.77	-.07	—											
3 Antiegalitarianism	2.96	1.27	.78	-.20***	.46***	—										
4 Opposition to equality	3.36	1.54	.88	.09*	.41***	.31***	—									
5 DWB	4.28	1.30	.84	-.13**	.17***	-.04	.04	—								
6 CWB	3.27	1.23	.79	-.08	.50***	.31***	.32***	.20***	—							
7 Fear of death	4.07	1.90	.92	-.02	.05	-.07	.14***	.26***	.04	—						
8 Poverty threat	3.20	.94	.88	-.11**	-.04	-.14***	-.10*	.23***	-.04	.30***	—					
9 Inequality threat	3.06	.92	.89	-.06	-.05	-.16***	-.13**	.25***	-.02	.24***	.76"*	—				
10 SSES	5.28	1.72	—	.21***	.06	.02	.14***	-.01	.08	.03	-.29***	-.18***	—			
11 OSES	3.53	1.13	—	.19***	.09*	.03	.10*	-.05	.05	-.03	-.33***	-.22***	.42***	—		
12 Gender	—	—	—	.06	–.09*	-.13**	-.04	.01	-.13**	.11**	.10*	.08	.07	-.01	—	
13 Education	4.05	1.22	—	.02	.01	.06	-.02	-.05	-.05	.01	-.04	-.01	.14***	.21***	.11*	—

Note. * p < .05, ** p < .01, *** p < .001. Gender (1 = “Male”, 2 = “Female”). CWB – Belief in Competitive world. DWB − Belief in dangerous world. OSES – objective socio-economic status. SSES – subjective socio-economic status.

Economic system justification was negatively associated with the belief in a dangerous world and the threat of poverty. Opposition to equality was positively associated with the belief in a competitive world and the fear of death, but negatively with economic threats. Similar associations were found regarding support for antiegalitarianism, except for its relation to the fear of death. Preferences for dominance were positively associated with the belief in a dangerous world and belief in a competitive world. SSES and OSES were positively associated with most system-justifying beliefs. In contrast to men, women showed less support for antiegalitarianism and dominance.

### Economic threats, the fear of death, dangerous and competitive worldview in supporting system-justifying beliefs

**Figure 1:**
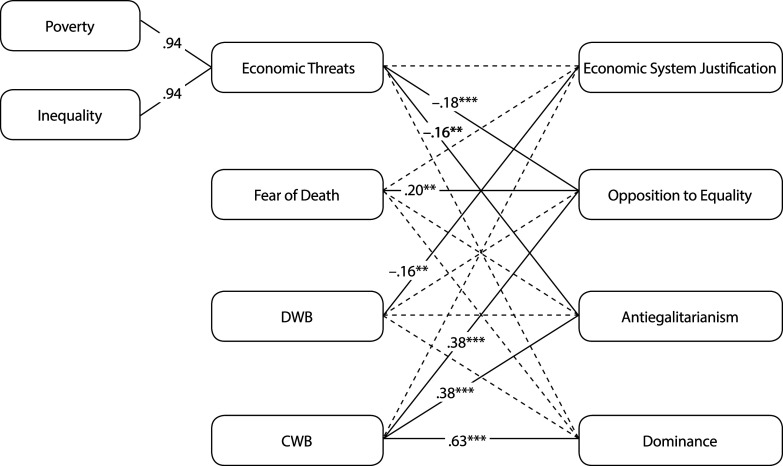
The role of threats in the support of system-justifying beliefs

The results of the SEM are presented in *Table 2* and *Figure 1*. The SEM model showed an acceptable fit to the data (*χ^2^*(854) = 1801; *RMSEA* = .044 [.041; .047]; *SRMR* = .055; *TLI* = .912; *CFI* = .920).

**Table 2:** Contribution of threats to the support of system-justifying beliefs

Variables		Dominance			Antiegalitarianism			Economic system justification			Opposition equality		
	B	SE	β_std_	B	SE	β_std_	B	SE	β_std_	B	SE	β_std_
Economic threats	–.11	.07	–.07	–.11^**^	.04	–.16^**^	–.01	.03	–.01	–.33^***^	.09	–.18^***^
DWB	.01	.06	.01	–.04	.03	–.07	–.09^**^	.03	–.16^**^	–.07	.08	–.05
CWB	.96^***^	.12	.63^***^	.26^***^	.06	.38^***^	–.04	.03	–.06	.69^***^	.11	.38^***^
Fear of death	.05	.03	.07	.00	.01	.01	.00	.01	.01	.16^***^	.04	.20^***^
OSES	.04	.05	.03	–.01	.02	–.01	.06^*^	.03	.12^*^	.05	.06	.03
SSES	–.02	.03	–.02	–.01	.02	–.02	.05^**^	.02	.10^**^	.07	.04	.05
Gender	–.03	.10	–.03	–.04	.05	–.08	.03	.05	.06	–.01	.12	–.01
Education	.02	.04	.01	.04^*^	.02	.07^*^	–.03	.02	–.05	–.04	.05	–.03
R^2^		.42			.18			.10			.20	

Note. B – unstandardized coefficient, SE – standard error, β– standardized coefficient. ^*^ p < .05, ^**^ p < *_std_* .01, _***_ p < .001. Gender (1 = “Male”, 2 = “Female”). CWB – Belief in competitive world. DWB − Belief in dangerous world. OSES – objective socio-economic status. SSES – subjective socio-economic status.

Belief in a competitive world positively predicted dominance (*β_std_* = .63; *z* = 8.23; *p* < .001), antiegalitarianism (*β_std_* = .38; *z* = 4.51; *p* < .001), opposition to equality (*β_std_* = .38; *z* = 6.55; *p* < .001). Belief in a dangerous world negatively predicted economic system justification (*β_std_* = –.16; *z* = –2.73; *p* = .006). Fear of death positively predicted opposition to equality (*β_std_* = .20; *z* = 4.24; *p* < .001). Economic threats (poverty and inequality) negatively predicted antiegalitarianism (*β_std_* = –.16; *z* = –2.84; *p* = .005) and opposition to equality (*β_std_* = –.18; *z* = –3.62; *p* < .001). Thus, results indicate that economic threats, fear of death, dangerous and competitive worldviews make significant and independent contributions to the support of system-justifying beliefs.

The role of socio-demographic variables in the support of system-justifying beliefs was consistent with previous studies. Higher educational level enhanced the support for antiegalitarianism, while higher subjective and objective socio-economic status increased the endorsement of economic system justification (Agadullina et al., 2021; Jost et al., 2017).

## Discussion

The aim of this study was to analyze the role of economic threats, the fear of death, belief in a dangerous world, and belief in a competitive world in the support of system-justifying beliefs. The results showed that belief in a competitive world made the most significant contribution to the support of system-justifying beliefs, in contrast to beliefs in a dangerous world, economic threats, and fear of death.

Belief in a competitive world enhanced the endorsement of dominance, antiegalitarianism, and opposition to equality. Individuals who viewed the world as a field of struggle for survival often perceive hierarchy and order as a way of organizing social relations (Jost, 2021). A competitive worldview oversimplifies social relations, portraying high status as enhancing the probability of *survival* through access to resources to *defend* against economic threats.

Concurrently, belief in a dangerous world negatively predicted economic system justification. Belief in a dangerous world enhanced the perception that the socioeconomic system threatens well-being and deemed unfair and unsafe. To recover the feeling of security and predictability, people may demonstrate support for beliefs that might restore the perceived control and order (Jost, 2021). For instance, under threat conditions, government regulation is perceived as a guarantor of the maintenance of social order, control, and stability (Jost, 2021; Prusova & Gulevich, 2020).

Moreover, the results showed that belief in a dangerous world and belief in a competitive world represent difierent forms of subjective threat, which have opposite effects on the support for system-justifying beliefs. The potential explanation might be that the perception of the world as generally unsafe represents a non-subjective form of threat, whereas belief in a competitive world implies the presence of a threatening other, that is, someone with whom one must compete for economic resources. For example, encountering economically advantaged outgroups may pose realistic threats, which can, in turn, enhance belief in the competitive nature of reality (Maddux et al., 2008).

The fear of death positively predicted opposition to equality. Support for unequal income distribution might be related to the recovery of perceived control through external attributions for success in life and the existing socio-economic order (Goode et al., 2014). For instance, previous research has found that, in Russia, reminders of death led to the support for control across various areas of social life (Prusova & Gulevich, 2020). Egalitarianism as a societal norm might be considered part of a cultural worldview that becomes polarized under threat. Terror Management Theory posits that polarization of pre-existing values, norms, and standards mitigates the effect of mortality salience (Greenberg et al., 1997).

Economic threats (socio-economic inequality and poverty) might reflect the disadvantaged conditions within the socio-economic system, leading people to attribute negative consequences to the system and, as a result, to perceive it as unfair (Kahn et al., 2022). The results showed that economic threats negatively predicted antiegalitarianism and opposition to equality. To deal with economic threats, people might delegate responsibility for meeting basic needs and ensuring safety to the state. Previous studies showed that economic crises enhanced trust in authoritarian political leaders, support for politicians with a military agenda, and preferences for political control (Torres-Vega et al., 2021). To analyze the difierences in defence mechanisms for societal threats, further studies should include system-justifying beliefs, the status quo, and control in the analysis.

Contradictory results regarding the effects of economic threats and fear of death illustrate the specifics of defence mechanisms through which system-justifying beliefs perform a palliative function. The effect of external threats on the system-justifying beliefs might be mediated by the subjective perception of the threats (e.g., belief in a dangerous world, belief in a competitive world), whereas internal threats might be mediated by restoring a sense of control (e.g., self-esteem) (Fritsche et al., 2008). For instance, in Spain the economic crisis enhanced the support for authoritarian leaders through the perception of the world as chaotic and fearful (Torress-Vega et al., 2021). In the United States, the effect of death reminders on the polarization of national identification was found to be mediated by self-esteem (Hohman & Hogg, 2015).

## Conclusion

Our research showed that system-justifying beliefs were dependent on economic threats, dangerous and competitive worldviews, and a fear of death. Belief in a competitive world had the most significant contribution to the preference for system-justifying beliefs. In line with SJT, system-justifying beliefs served a palliative function by restoring the perception of control and order in social relations. Finally, further studies need to clarify the causal relations between difierent types of threats and system-justifying beliefs and ideologies, such as maintenance of the status quo and economic inequality for groups with low and high socio-economic status.

## Limitations

Despite the significant contribution of the threats under study to the support of system-justifying beliefs, the current research has several limitations. The strong relationship between the social perception of the threats and system-justifying beliefs did not allow us to determine the causal effect. In line with Integrated Threat Theory, social attitudes towards the world and system-justifying ideologies can afiect perceptions of and behavioral responses to threats (Stephan & Renfro, 2002). For instance, previous studies suggested that conservatives might be more sensitive to negative stimuli, however, in other studies conservatives (high system justifiers) showed weaker emotional responses in comparison to liberals (low system justifiers) (Goudarzi et al., 2020). Further experimental research is recommended to test the causal effect of internal and external threats on system-justifying beliefs in the economic domain.

In the current study, the majority of participants had low incomes. Previous studies showed that people with low incomes are more sensitive to perceptions of and responses to internal and external threats (Kraus et al., 2012). To deal with negative views of the world, people with low socio-economic status tend to justify their position in the social hierarchy and support government regulation in the socio-economic sphere (e.g., equal income distribution) (Kraus et al., 2012). Hadarics et al. (2021) found that people with low socio-economic status who reported a low level of perceived control also tended to seek external sources to regain a sense of security and predictability in the social world, such as a strong government.

In the current model we analysed the direct contribution of difierent types of threat to the system-justifying beliefs in the socio-economic sphere. At the same time, the threats under study might reflect the difierent levels of analysis, ranging from external threats (macro-level) to subjective perception of threat or internal threats (micro-level), which may require the use of a model with a more complex structure. Furthermore, we drew on Jost et al.’s (2017) model, which suggests an association between existential needs, consisting of internal, external threats and subjective perceptions of threats, and support of system-justifying beliefs. However, in the current study, the threats within the common cluster, *e.g*., subjective perceptions of threat, demonstrated specific associations with support for system-justifying beliefs in the economic domain. For example, belief in a dangerous world negatively predicted economic system justification, while belief in a competitive world showed no significant association. This pattern highlights the need to develop alternative classification of perceived threats in the Russian context in the future studies.
